# Maternal and Perinatal Outcomes of Twin Pregnancy in 23 Low- and Middle-Income Countries

**DOI:** 10.1371/journal.pone.0070549

**Published:** 2013-08-01

**Authors:** Joshua P. Vogel, Maria Regina Torloni, Armando Seuc, Ana Pilar Betrán, Mariana Widmer, João Paulo Souza, Mario Merialdi

**Affiliations:** 1 School of Population Health, Faculty of Medicine, Dentistry and Health Sciences, University of Western Australia, Perth, Western Australia, Australia; 2 UNDP/UNFPA/UNICEF/WHO/World Bank Special Programme of Research, Development and Research Training in Human Reproduction, Department of Reproductive Health and Research, World Health Organization, Geneva, Switzerland; 3 Department of Obstetrics, São Paulo Federal University, São Paulo, Brazil; 4 Brazilian Cochrane Centre, São Paulo, Brazil; Aga Khan University, Pakistan

## Abstract

**Background:**

Twin pregnancies in low- and middle-income countries (LMICs) pose a high risk to mothers and newborns due to inherent biological risks and scarcity of health resources. We conducted a secondary analysis of the WHO Global Survey dataset to analyze maternal and perinatal outcomes in twin pregnancies and factors associated with perinatal morbidity and mortality in twins.

**Methods:**

We examined maternal and neonatal characteristics in twin deliveries in 23 LMICs and conducted multi-level logistic regression to determine the association between twins and adverse maternal and perinatal outcomes.

**Results:**

279,425 mothers gave birth to 276,187 (98.8%) singletons and 6,476 (1.2%) twins. Odds of severe adverse maternal outcomes (death, blood transfusion, ICU admission or hysterectomy) (AOR 1.85, 95% CI 1.60–2.14) and perinatal mortality (AOR 2.46, 95% CI 1.40–4.35) in twin pregnancies were higher, however early neonatal death (AOR 2.50, 95% CI 0.95–6.62) and stillbirth (AOR 1.22, 95% CI 0.58–2.57) did not reach significance. Amongst twins alone, maternal age <18, poor education and antenatal care, nulliparity, vaginal bleeding, non-cephalic presentations, birth weight discordance >15%, born second, preterm birth and low birthweight were associated with perinatal mortality. Marriage and caesarean section were protective.

**Conclusions:**

Twin pregnancy is a significant risk factor for maternal and perinatal morbidity and mortality in low-resource settings; maternal risk and access to safe caesarean section may determine safest mode of delivery in LMICs. Improving obstetric care in twin pregnancies, particularly timely access to safe caesarean section, is required to reduce risk to mother and baby.

## Introduction

The World Health Organization (WHO) estimates that 99% of the world's annual 287,000 annual maternal deaths and 3 million neonatal deaths occur in developing countries. [1], [2] Due to inherent biological factors, twin pregnancies have increased rates of obstetric and perinatal complications such as preeclampsia, post-partum haemorrhage and preterm birth [3]–[6], which are known risk factors for maternal and perinatal mortality. Multiple pregnancies in low-resource settings pose higher feto-maternal risks due to a scarcity of human and material resources, which translate into insufficient care during pregnancy and delivery. This is particularly true of central African countries with high twinning rates and limited health infrastructure. [3] Therefore, multiple pregnancies in developing countries expose mother and infants to extremely high risks. As multiple births can contribute significantly to maternal and perinatal morbidity and mortality, it is important to investigate the magnitude of the increase in feto-maternal risk. The outcome of twin pregnancies across developing countries has not been extensively investigated.

Twin birth registries are rare in low- and middle-income countries (LMICs) [4] and twin-specific research is generally on hospital-based cohort studies, secondary analyses of interventional trials or retrospective analyses of Demographic and Health Survey (DHS) data, all with significant limitations and bias. [3], [5]–[9] Data are particularly limited on maternal outcomes from twin births. Studies on twin pregnancies in LMICs are difficult and expensive to conduct. The WHO Global Survey on Maternal and Perinatal Health (WHOGS) offers the possibility of analyzing the maternal and perinatal outcomes of twin compared to singleton pregnancies in 23 countries.

To address the limitations in the literature we analyzed maternal and perinatal outcomes in twins compared to singletons in the WHOGS dataset. We also examined factors associated with foetal/neonatal morbidity and mortality in twin pregnancies.

## Methods

The WHO Global Survey on Maternal and Perinatal Health (WHOGS) is a multi-country, cross-sectional survey of all women delivering in participating health institutions over a two or three-month period (depending on volume of deliveries). Data on over 290,000 women in 373 randomly selected institutions across 24 countries in Africa, Latin America and Asia were collected. The WHOGS was conducted over 2004–2005 (Africa and Latin America) and 2007–2008 (Asia).

The methodological details of the survey have been published previously. [10], [11] In brief, a stratified, multistage sampling design was used to obtain a global sample of countries and health institutions. Two randomly selected provinces and the capital city were sampled from each country. Within these, seven institutions were randomly selected from a census of institutions with over 1,000 births per year and able to perform caesarean sections. All women who delivered after 20 weeks gestation during the data collection period in participating institutions were included. Women who died before delivery or following a postpartum referral were excluded, as were maternal or infant deaths that took place after discharge or the seventh postpartum day. Trained data collectors reviewed medical records during the study period and used this data to complete the individual data form.

For this study we analyzed maternal and perinatal data from singleton and twin pregnancies in 23 LMICs (Japan was excluded as it is a high-income country). The primary maternal outcome was maternal death (death before seven days postpartum or discharge), and we used a severe adverse maternal outcome index (maternal death, blood transfusion, admission to ICU or hysterectomy) as a secondary maternal outcome. This index has been validated in previous WHOGS analyses. [12] The perinatal outcomes were stillbirth (an infant born with no signs of life), early neonatal death (liveborn neonate that died in the first seven days of life, prior to discharge) and perinatal mortality (stillbirth and early neonatal death). Early neonatal deaths occurring after discharge and the chorionicity of multiple pregnancies were not captured in the WHOGS dataset.

We initially conducted a descriptive analysis of maternal and fetal characteristics in twin versus singleton pregnancies. Multilevel logistic regression was performed (using the LMER procedure in R) to determine the adjusted odds of maternal and perinatal outcomes associated with twin pregnancies. Models accounted for the clustering of mothers within facilities (for both perinatal and maternal outcomes) and the clustering of twins within mothers (for perinatal outcomes). Clustering of twins within mothers is often overlooked and can lead to over-estimation of effect if neglected. [13] For the model of maternal outcomes, we adjusted for covariates based on variables available in the WHOGS dataset, clinical and epidemiological evidence of confounding effects in the literature and results of the bivariate analysis: maternal age (<18, 18–35, >35), education (0, 1–4, 5–9, > = 10 years), marital status, parity (0, 1–2, > = 3), number of antenatal visits (0, 1–3, > = 4), medical conditions (chronic hypertension, malaria), obstetric antenatal conditions (prelabour rupture of membranes, pregnancy-induced hypertension, pre-eclampsia, eclampsia, vaginal bleeding in the second half of pregnancy), mode of delivery (vaginal or caesarean section) and facility (as a random effect). For modelling perinatal outcomes, we adjusted for the same maternal-level variables and also relevant neonatal-level variables, namely birth order, infant sex and fetal presentation. Missing values were reported, however models were based on completed cases only (missing values excluded). To determine risk factors for perinatal mortality amongst twins only, we determined odds ratios for maternal demographical factors, medical and obstetric conditions, delivery characteristics and neonatal factors.

We considered p-values<0.05 to be statistically significant. Statistical analysis was performed using SPSS (PASW Statistics 20, Chicago, USA) and R (R v2.14.2, Vienna, Austria). [14], [15] Ethical clearance from all Ministries of Health of participating countries, WHO Ethics Review Committee and that of each health institution or sub-region were obtained.

## Results

The WHOGS dataset contains 290,610 deliveries in 24 countries. We excluded deliveries in Japan (n = 3,356), neonates with congenital malformations (n = 3,429) and deliveries with missing data on birth order, number of neonates or congenital malformations (n = 953). We also excluded higher-order multiple births (n = 207) as it was beyond the scope of this analysis. We analysed data from 279,425 mothers delivering 282,663 newborns –276,187 (98.8%) singletons and 6,476 (1.2%) twins. Rates of missing data were <1% for all variables, except for maternal education (4.9%) and antenatal care (5.7%). The highest twin rates in this dataset were in Nigeria (4.2% of births), the Democratic Republic of Congo (3.8%) and Niger (3.6%).

Tables 1 and 2 describe maternal characteristics, mode of delivery and maternal outcomes of twin versus singleton pregnancies. Differences in maternal characteristics were slight yet significant – mothers of twins were older, less educated, of higher parity and received slightly less antenatal care. They generally had more medical and obstetric complications of pregnancy and delivery (except for HIV, pregestational diabetes and pyelonephritis/UTI). The rate of caesarean section was significantly higher in twin pregnancies (42.9% vs 25.5%).

**Table 1:** Characteristics of twin and singleton pregnancies in 23 low- and middle-income countries.

	Mothers of Twins N = 3,238	Mothers of Singletons N = 276,187	Chi-square P-value
**Maternal Age**			
<18	76 (2.3)	13,110 (4.8)	<0.001
18–35	2,828 (87.3)	240,314 (87.0)	
>35	332 (10.3)	22,125 (8.0)	
Missing	2 (0.1)	638 (0.2)	
**Years of Education**			
0	333 (10.3)	20,631 (7.5)	<0.001
1–4	192 (5.9)	16,210 (5.9)	
5–9	1,213 (37.5)	103,142 (37.3)	
> = 10	1,309 (40.4)	122,755 (44.4)	
Missing	191 (5.9)	13,449 (4.9)	
**Married**	2,911 (89.9)	237,325 (85.9)	<0.001
**Parity**			
Parity 0	1,109 (34.2)	118,228 (42.8)	<0.001
Parity 1–2	1,405 (43.4)	118,656 (43.0)	
Parity > = 3	702 (21.7)	38,413 (13.9)	
**Number of antenatal care visits**			
0	188 (5.8)	12,700 (4.6)	<0.001
1–3	815 (25.2)	67,271 (24.4)	
4 or more	1,993 (61.5)	180,457 (65.3)	
Missing	242 (7.5)	15,759 (5.7)	
**Medical history**			
HIV	28 (0.9)	2,233 (0.8)	0.69
Chronic hypertension	44 (1.4)	2,306 (0.8)	<0.001
Malaria	152 (4.7)	7,259 (2.6)	<0.001
Pregestational diabetes	20 (0.6)	1,896 (0.7)	0.64
Pyelonephritis/UTI	227 (7.0)	18,058 (6.6)	0.28
**Complications in current pregnancy**	402 (12.4)	28,089 (10.2)	<0.001
Prelabour rupture of membranes	271 (8.4)	10,249 (3.7)	<0.001
Pregnancy-induced hypertension	245 (7.6)	8,304 (3.0)	<0.001
Pre-eclampsia/eclampsia	35 (1.1)	1,507 (0.5)	<0.001
Severe anaemia*	67 (2.1)	3,603 (1.3)	<0.001
Vaginal bleeding in 2^nd^ half of pregnancy	402 (12.4)	28,089 (10.2)	<0.001
**Mode of Delivery**			
Vaginal delivery	1,849 (57.1)	205,508 (74.4)	<0.001
Caesarean section	1,389 (42.9)	70,399 (25.5)	
Missing	0 (0.0)	280 (0.1)	
Spontaneous vaginal delivery	1,797 (55.5)	200,645 (72.6)	<0.001
Instrumental vaginal delivery	52 (1.6)	4,863 (1.8)	
Elective (no labour) caesarean section	506 (15.6)	22,835 (8.3)	
Other types of caesarean section**	883 (27.3)	47,564 (17.2)	

* Severe anaemia: Haemoglobin <7 g/L at any time during pregnancy.

** Emergency or intrapartum caesarean section.

**Table 2:** Maternal outcomes in twin and singleton deliveries.

	Mothers of Twins N = 3,238	Mothers of Singletons N = 276,187	Chi-square P-value
Maternal death	9 (0.3)	326 (0.1)	0.009
Blood transfusion	161 (5.0)	4,268 (1.6)	<0.001
Admission to ICU	188 (5.8)	5,912 (2.1)	<0.001
Hysterectomy	8 (0.3)	323 (0.1)	0.032
**Severe Adverse Maternal Outcome Index** * **:**			
All deliveries	309 (9.6)	9,655 (3.5)	<0.001
Vaginal deliveries only	104 (5.6)^a^	3,070 (1.5)^a^	<0.001
Caesarean deliveries only	205 (14.8)^b^	6,572 (9.3)^b^	<0.001

* Defined as maternal death, blood transfusion, admission to ICU or hysterectomy.

a Denominator is vaginal deliveries only: 1,849 for twins and 205,508 for singletons.

b Denominator is caesarean deliveries only : 1,389 for twins and 70,399 for singletons.

The prevalence of maternal death was higher in twin mothers compared to singleton mothers (0.3% vs 0.1%, p<0.009), although there were only 9 maternal deaths in twin pregnancies. Severe adverse maternal outcomes were more common in twin pregnancies (9.6% vs 3.5%, p<0.001), a pattern which persisted when stratified by region (data not shown). When the severe adverse maternal outcome index was stratified by mode of delivery (Table 2), more cases occurred following caesarean section. For all women undergoing caesarean section, the prevalence of severe adverse maternal outcome was higher for mothers of twins than singletons (14.8% vs 9.3%, p<0.001). This relationship held at the regional level for Latin America (7.9% vs 4.0%) and Asia (15.0% vs 8.9%), however in Africa severe adverse maternal outcome following caesarean was 30.2% for mothers of twins and 29.1% for mothers of singletons.

Table 3 shows significantly higher rates of preterm birth, low birth weight and perinatal outcomes in twin deliveries. These patterns persisted regardless of region, mode of delivery or gestational age (term vs preterm) (data not shown).

**Table 3:** Perinatal characteristics and outcomes in twin and singleton pregnancies.

	Twins N = 6,476	Singletons N = 276,187	Chi-square P-value^a^
Mean gestational age (SD)	36.8 (3.0)	38.7 (2.1)	<0.001
Preterm birth (<37 weeks)	2,277 (35.2)	26,645 (9.6)	<0.001
Early preterm birth (< = 32 weeks)	396 (6.1)	3,760 (1.4)	<0.001
Moderate preterm birth (32–33 weeks)	378 (5.8)	2,944 (1.1)	<0.001
Late preterm birth (34 – <37 weeks)	1,503 (23.2)	19,941 (7.2)	<0.001
Missing GA	105 (1.6)	3,362 (1.2)	
Mean birth weight (SD)	2,351.9 (617.3)	3,094.7 (548.5)	<0.001
Low birth weight (<2500 g)	30 (0.5)	550 (0.2)	<0.001
Missing birthweight	30 (0.5)	550 (0.2)	
Small for gestational age^b^	2,487 (38.4)	26,676 (9.7)	<0.001
5 minute APGAR<7	703 (10.9)	11,371 (4.1)	<0.001
Missing APGAR	44 (0.7)	1,867 (0.7)	
Admission to NICU	1,873 (28.9)	24,283 (8.8)	<0.001
Stillbirth	259 (4.0)	4,741 (1.7)	<0.001
Early neonatal death	202 (3.1)	1,764 (0.6)	<0.001
Perinatal mortality^c^	461 (7.1)	6,505 (2.4)	<0.001

a P-value adjusted for clustering effect of mothers in twin deliveries [13].

b Definition of small-for-gestational-age based on global reference described by Mikolajczyk and colleagues [25].

c Perinatal mortality defined as stillbirth and early neonatal death.

Table 4 shows crude and adjusted odds ratios for maternal and perinatal outcomes associated with twins. Maternal death was not considered as there were too few cases for modelling. Regardless of mode of delivery, mothers of twins had increased odds of severe adverse outcome (AOR 1.85, 95% CI 1.60–2.14). When stratified by mode of delivery, odds of severe adverse outcome were higher for mothers who delivered vaginally (AOR 2.97, 95% CI 2.38–3.70) than by caesarean (AOR 1.68, 95% CI 1.36–2.08). The odds of stillbirth (AOR 1.22, 95% CI 0.58–2.57) and early neonatal death (AOR 2.50, 95% CI 0.95–6.62) in twins did not reach statistical significance, however odds of perinatal mortality were significantly higher (AOR 2.46, 95% CI 1.40–4.35).

**Table 4:** Crude and adjusted odds ratios for adverse maternal and perinatal outcomes in twins versus singleton pregnancies.

	Crude OR (95% CI)	Adjusted OR (95% CI)
Severe adverse maternal outcome index^1^	2.91 (2.59–3.29)	1.85 (1.60–2.14)*
Severe adverse maternal outcome index^1^ in Vaginal deliveries only	3.94 (3.22–4.81)	2.97 (2.38–3.70)*
Severe adverse maternal outcome index^1^ in caesarean deliveries only	1.69 (1.45–1.96)	1.68 (1.36–2.08)*
Stillbirth	2.39 (2.10–2.71)	1.22 (0.58–2.57)†
Early neonatal death	5.15 (4.44–5.95)	2.50 (0.95–6.62)†
Perinatal mortality	3.18 (2.88–3.51)	2.46 (1.40–4.35)†

1 Severe adverse maternal outcome index: maternal death, admission to ICU, blood transfusion or hysterectomy.

* Odds ratio adjusted for maternal age, maternal education, marital status, parity, antenatal visits, malaria, prelabour premature rupture of membranes, chronic hypertension, pregnancy-induced hypertension, pre-eclampsia, eclampsia, vaginal bleeding in 2^nd^ half of pregnancy, mode of delivery and facility (as random effect).

† odds ratio adjusted for maternal age, maternal education, marital status, parity, antenatal visits, mode of delivery, chronic hypertension, malaria, prelabour premature rupture of membranes, pregnancy-induced hypertension, pre-eclampsia, eclampsia, vaginal bleeding in 2^nd^ half of pregnancy, severe anaemia, infant sex, birth order, fetal presentation, facility (as a random effect) and clustering effect of twin neonates.

Of the 6,476 twin neonates, 461 (7.1%) experienced perinatal mortality while 6,001 (92.7%) did not (14 twin neonates had missing data for these outcomes and were excluded). (Table 5) Maternal age less than 18, less than 10 years education, less than 4 antenatal care visits, nulliparity and vaginal bleeding in the second half of pregnancy were associated with perinatal mortality. Being married and delivery by caesarean section were associated with better perinatal outcomes. Twins in a non-cephalic presentation, born second, preterm or low birthweight were also associated with perinatal mortality, as was being the smaller twin of a birthweight discordant pair. A multilevel logistic regression model of the twin-only population was underpowered and could not provide any meaningful data.

**Table 5:** Factors associated with adverse perinatal outcomes in twin pregnancies in 23 low- and middle-income countries.

	Perinatal mortality^a^ N = 461	Perinatal survival N = 6,001	Crude OR (95% CI)
**Maternal Age**			
<18	24 (5.2)	127 (2.1)	2.55 (1.63–4.00)
18–35	389 (84.4)	5,257 (87.6)	Reference
>35	48 (10.4)	613 (10.2)	1.06 (0.78–1.45)
Missing	0 (0.0)	4 (0.1)	
**Years of Education**			
0	79 (17.1)	585 (9.7)	2.82 (1.71–3.04)
1–4	35 (7.6)	347 (5.8)	1.70 (1.16–2.51)
5–9	176 (38.2)	2,245 (37.4)	1.33 (1.06–1.66)
> = 10	146 (31.7)	2,467 (41.1)	Reference
Missing	25 (5.4)	357 (5.9)	
**Marital status**			
Married	395 (85.7)	5,417 (90.3)	Reference
Not married	64 (13.9)	562 (9.4)	1.56 (1.18–2.06)
Missing	2 (0.4)	22 (0.4)	
**Parity**			
Parity 0	193 (41.9)	2,020 (33.7)	1.46 (1.18–1.81)
Parity 1–2	172 (37.3)	2,633 (43.9)	Reference
Parity > = 3	94 (20.4)	1,306 (21.8)	1.10 (0.85–1.43)
Missing	2 (0.4)	42 (0.7)	
**Antenatal care visits**			
0	52 (11.3)	323 (5.4)	3.28 (2.36–4.55)
1–3	184 (39.9)	1,444 (24.1)	2.60 (2.10–3.21)
4 or more	186 (40.3)	3,789 (63.1)	Reference
Missing	39 (8.5)	445 (7.4)	
**Medical history**			
HIV	0 (0.0)	54 (0.9)	*
Chronic hypertension	5 (1.1)	83 (1.4)	0.78 (0.32–1.94)
Malaria	22 (4.8)	282 (4.7)	1.02 (0.65–1.59)
Pregestational diabetes	0 (0.0)	40 (0.7)	*
Pyelonephritis/UTI	28 (6.1)	425 (7.1)	0.85 (0.57–1.26)
**Complications in current pregnancy**			
Prelabour rupture of membranes	57 (12.4)	745 (12.4)	1.00 (0.75–1.33)
Pregnancy-induced hypertension	24 (5.2)	518 (8.7)	0.58 (0.38–0.89)
Pre-eclampsia/eclampsia	31 (6.7)	457 (7.6)	0.88 (0.60–1.28)
Severe anaemia	7 (1.5)	63 (1.1)	1.46 (0.66–3.19)
Vaginal bleeding in 2^nd^ half of pregnancy	17 (3.7)	117 (2.0)	1.93 (1.15–3.24)
**Mode of delivery**			
Vaginal delivery	343 (74.4)	3,326 (55.4)	Reference
Caesarean section (any type)	118 (25.6)	2,673 (44.5)	0.43 (0.35–0.53)
Non-cephalic presentation	172 (37.3)	1,744 (29.1)	1.46 (1.20–1.78)
Born second	269 (58.4)	2,963 (49.4)	1.44 (1.19–1.74)
Birth weight discordance^b^	132 (28.6)	831 (13.8)	2.61 (2.09–3.26)
Male gender	249 (54.5)	3,043 (50.7)	1.16 (0.96–1.41)
Preterm birth (<37 weeks)	295 (64.0)	1,973 (32.9)	3.98 (3.24–4.89)
Low birth weight (<2500 g)	392 (85.0)	3,172 (52.9)	6.10 (4.60–8.08)

a Perinatal mortality defined as stillbirth or early neonatal death.

b smaller twin in a birth weight discordant (>15%) twin pair (Weight of larger twin – weight of smaller twin)/weight of larger twin ] * 100.

* unable to calculate OR as zero cases in perinatal mortality group.

## Discussion

The most significant findings were the increased prevalence and adjusted odds of severe adverse maternal outcomes (AOR 1.85, 95% CI 1.60–2.14) and perinatal mortality (AOR 2.46, 95% CI 1.40–4.35) in twin pregnancies compared to singleton pregnancies, regardless of mode of delivery. This suggests that in LMICs a twin pregnancy, or being a twin, confers an intrinsic risk of severe adverse outcomes at both the maternal and neonatal level. These findings are congruent with previous literature, [16]–[19] however the extent to which this risk was due to environmental factors in low-resource settings (such as a lack of appropriate antenatal or intrapartum care) or the intrinsic risk of twin pregnancy itself remains unclear.

Mothers of twins in LMICs were slightly older, which may partially explain higher rates of medical conditions and antenatal complications. Lower rates of education and antenatal visits and higher rates of malaria may reflect the higher prevalence of twin pregnancies in lower-resource settings. We had difficulty determining whether vaginal bleeding in the second half of pregnancy was an appropriate confounder in the stillbirth model, as it may potentially lie in the causal pathway. However, when we excluded it as a confounder on sensitivity analysis, it made very little difference to the adjusted odds ratio estimate.

As we expected, for both twins and singletons the prevalence of severe adverse maternal outcome was higher following caesarean compared to vaginal deliveries, likely due to the indications for caesarean and post-surgical management (ie: admission to ICU). However, the odds of severe adverse maternal outcomes were more pronounced in vaginal delivery (AOR 2.97, 95% CI 2.38–3.70) then in caesarean section (AOR 1.68, 95% CI 1.36–2.08). This suggests that maternal risk in twin pregnancy may be partially mitigated by caesarean section, even though the prevalence of severe outcome for the mother is higher overall following caesarean due to the underlying indication.

However, when stratified by region, prevalence of severe adverse maternal outcomes following caesarean for twins versus singletons varied widely. In Africa, the prevalence of severe adverse maternal outcomes was significantly higher following caesarean for both twin pregnancies (30.2%) and singletons (29.1%). This may be due to poorer access to safe caesarean section (a known issue in many LMICs [20]) and comprehensive emergency obstetric care, or patients presenting at a later, more complicated stage. In Latin America, caesarean use is more widespread [21] and severe adverse maternal outcomes in both twin and singleton pregnancies were lower (7.9% and 4.0%). The risk of adverse maternal outcomes following caesarean in twin pregnancies is seemingly dependent on contextual factors. While the debate surrounding appropriate mode of twin delivery is driven more by the risk to fetus rather than the risk to the mother, [22] timely access to safe caesarean section in the event of complications is critical to the mother’s health as well. In areas where safe caesarean section is not available, the risk to mother must be carefully considered.

Obstetric care providers in low-resource settings should consider factors associated with perinatal mortality in twins when catering to twin pregnancies. The seemingly protective association with pregnancy-induced hypertension is likely driven by pregnancies that ended in stillbirth that do not have the opportunity to develop this condition. The decreased odds of perinatal mortality associated with caesarean section (OR 0.43, 95% CI 0.35–0.53) supports the view that caesarean mitigates risk in twin pregnancies in LMICs, however multilevel logistic regression within the twin neonate population was not sufficiently powered to determine if this is an independent effect.

This study addresses a significant gap in the literature on the outcomes of twin deliveries in low- and middle-income countries, where the frequency of twin birth is often high and epidemiological research on multiple births is challenging. To the best of our knowledge, it is the largest study on this topic to date. Previous studies of mode of delivery in twin pregnancy have been confined to high-income countries, where rates of severe adverse maternal outcomes are lower than in LMICs and severe maternal morbidity and mortality are not routine primary outcomes. [22] The few existing studies on twin pregnancies in LMICs come from single institutions in a few countries and report on sample sizes of only 100 to 400 twin neonates [5], [6], [8], [9] which hinders in-depth analyses of their characteristics and outcomes compared to singletons. The largest study on twin pregnancies in LMICs presented prevalence and trends of twinning in 75 low and middle-income countries, using data from demographic and health surveys held in the last two decades [3].

This study has limitations that should be noted. We did not have sufficient numbers of maternal deaths in twin pregnancies to use it as a primary outcome for modelling. We lacked data on several maternal and neonatal variables, such as race, smoking status and household income, and data was abstracted from medical records rather than from direct patient interview. Therefore, despite the prospective nature of the study and the involvement of personnel specifically trained for this study, this led to missing information especially on maternal education and number of antenatal care visits. In some LMICs, medical record documentation is suboptimal, which may have affected data quality. We had no information on chorionicity or use of assisted reproductive technologies, which are known to have a significant impact on fetal and neonatal outcomes. [23] Our analysis did not explore interaction of variables or effect modification. As a descriptive analysis of deliveries in larger, referral facilities in LMICs, these results cannot be generalised to women in community settings or to those delivering at home, which in some African countries represents more than half of all deliveries. [24] It can be inferred that the maternal and perinatal risks of twin deliveries in these settings will probably be higher than those currently presented.

### Conclusion

Our findings suggest that maternal and perinatal morbidity and mortality associated with twin births in low-resource settings is significant, and twin pregnancy poses an intrinsic risk to both mothers and neonates. Despite the rate of severe adverse maternal outcomes following Caesarean section (CS) in twin pregnancies, there is some evidence that CS partially mitigates the risk of adverse maternal and perinatal outcomes compare to vaginal deliveries. However, in African countries adverse maternal outcomes following CS for twin pregnancies was significantly higher. In resource-constrained settings, both maternal risk and access to safe caesarean section must be carefully weighed when considering the safest mode of delivery.

Focused interventions to improve antenatal, delivery and postnatal care in twin pregnancies should be considered a priority in strategies to reduce overall morbidity and mortality in low-resource settings, including prioritizing timely access to safe CS for all mothers, particularly those with twin pregnancies. Further research to quantify the total burden of disease posed by twin pregnancies in LMICs and the role of caesarean section in their management would be of great benefit in making it a higher priority in the global maternal and perinatal health landscape.
